# Integrative proteomics and metabolomics of Guizhou Miao Sour Soup affecting simple obese rats

**DOI:** 10.3389/fnut.2022.1019205

**Published:** 2022-11-03

**Authors:** Qin Yuan, Qianqian Zhou, Nanlan Wang, Yuancui Zheng, Hua Hu, Shiyao Hu, Huiqun Wang

**Affiliations:** ^1^School of Public Health, The Key Laboratory of Environmental Pollution Monitoring and Disease Control, Ministry of Education, Guizhou Medical University, Guiyang, China; ^2^Guizhou Food Nutrition and Health Engineering Research Center, Guiyang, China

**Keywords:** Sour Soup, obesity, proteomics, metabolomics, lipids

## Abstract

Miao Sour Soup (MSS) is a fermented product from the Qiandongnan region of Guizhou Province, which enrich many beneficial ingredients and is widely consumed in the whole China. Fermented food is beneficial to physical health with the potential positive regulating affection on simple obesity. In this study, we analyzed the mechanism of action of MSS to prevent simple obesity induced by high-fat diet by proteomics and metabolomics. Quantitative proteomics with tandem mass tagging labeling and liquid chromatography-mass spectrometry was used to analyze the changes of liver proteins and metabolites after the MSS intervention. MSS intervention upregulated 33 proteins and 9 metabolites and downregulated 19 proteins and 10 metabolites. Bioinformatics analysis showed that MSS could prevent simple obesity by acting on the PPAR signaling pathway, retinol metabolism, fatty acid β-oxidation, fatty acid degradation, fatty acid biosynthesis, glycine, serine and threonine metabolism, pyruvate metabolism, citrate cycle (TCA cycle) and other signaling pathways. This study provides new insights into the use of MSS to prevent simple obesity caused by high-fat diets and the search for healthy eating patterns with MSS.

## Introduction

Simple obesity refers to the accumulation of excessive body fat, resulting in body mass index (BMI) value more than the normal level, the increasing proportion of high-calorie food intake is the main reason for the occurrence of simple obesity (1). Simple obesity can further lead to health risks such as insulin resistance, hyperlipidemia, and impaired immune function (2). The World Health Organization reported that obesity is the leading cause of disability and death in many parts of the world and that a sustainable approach to effective obesity prevention is a more affordable strategy from a public health perspective (3). Miao Sour Soup (MSS) is a common food with many years of history of consumption in the minority region of Qiandongnan, Guizhou Province, and is a fermented product made from tomatoes and red peppers after two fermentations (4). Previous research found that MSS contains citral, limonene, α-pinene, β-pinene, ethyl linoleate, ethyl linolenic acid, and other flavor components, but also contains lycopene and capsaicin and other bioactive ingredients (5). Studies have shown that lycopene enhances the body’s antioxidant function and has an ameliorative and preventive effect on chronic non-communicable diseases (6). Capsaicin is the active component of Capsicum annum, a plant of the genus Capsicum. It was found that capsaicin can activate the transient receptor potential cation channel subfamily V member 1 (TRPV1) pathway and increase the level of glucagon-like peptide 1 (GLP-1) in rats, thereby enhancing insulin action and inhibition of glucagon secretion to control diet-related glycemia. In addition, capsaicin is involved in peroxisome proliferator-activated receptor-γ (PPARγ) activation, thereby inhibiting the inflammatory response of macrophages in adipose tissue (7). Animal experiments found that limonene has antibacterial, antioxidant, anti-inflammatory, and anti-tumor effects (8), α-pinene and β-pinene can inhibit tumor growth and anti-inflammation, etc. (9). In the process of fermentation, acids such as lactic acid, acetic acid, and citric acid were also produced, which have the effects of regulating inflammation and promoting immune tolerance, neuroprotection, anti-aging, and improving ketosis in rats (10–12). Thus, there are several beneficial components present in MSS that may contribute to the health of the organism.

Previous studies found that the water extract of soy milk fermented with *Lactobacillus paracasei* subsp. *paracasei* NTU 101 (W101) could inhibit CCAAT/enhancer-binding protein β (C/EBPβ) expression, increase lipase activity and decrease heparin-releasable lipoprotein lipase activity, thereby reducing adipogenesis (13). Another study found that whey drinks fermented by lactic acid bacteria inhibited weight gain, organ weight gain, and white adipose tissue formation, reduced blood lipid and appetite-related hormone levels, and improved insulin sensitivity in obese rats on a high-fat diet (14). These findings suggest that fermented foods may have an ameliorative effect on obesity caused by a high-fat diet. With the development of the economy, the excessive intake of energy-dense foods in the dietary structure and the resulting increase in the prevalence of obesity is a public health problem in all regions of the world (15), imposing a disease burden on people in many countries and regions (3). Currently, finding new ways to prevent or treat obesity is a global concern, especially the prevention of simple obesity and lipid metabolism disorders through food itself is a hot research topic. These studies focused on foods and their extracts that reduce intake by inhibiting orexigenic signals or enhancing anorexigenic signals, limiting the bioavailability of nutrients (by inhibiting digestive enzymes and/or interacting with them to reduce their absorption), stimulating energy expenditure (EE) (thermogenesis), and altering the composition of the gut microbiota (16).

In our previous study, we observed that intervention with MSS reduced obesity caused by a high-fat diet, lowered elevated blood triglycerides, cholesterol and LDL cholesterol, and had an ameliorating effect on inflammatory factors such as TNF-α and IL-6 *in vivo*, as well as on their intestinal flora (17, 18). However, we conducted further experiments and found that similar intervention effects could not be achieved in obese rats by intervening with any single component of the main functional components of MSS, such as lycopene, capsaicin or organic acids. The current view of food histology emphasizes that the research process should focus on the effect of food as a whole on the health of the organism, rather than the effect of a single component of food on health (19). It has been extended from the traditional field of studying only the analysis of nutrient composition in food, the biochemical metabolism and physiological functions of nutrients in the human body, and the risks of nutrient deficiency or excess on human health to the field of how to balance the nutritional system in the body through dietary regulation to maintain the health of the body and prevent the occurrence of certain nutrition-related diseases (20). Therefore, we propose the hypothesis that MSS works not based on a single component, but on a special “food ecology” formed by the combination of its rich bioactive substances and the beneficial components produced during the fermentation process, and that its health effects are the result of the combined action of such a “food ecology.” Proteomics and metabolomics techniques enable the systematic measurement of overall protein and metabolite changes in various physiological and pathological processes (21, 22). The liver is the regulatory center of the body’s metabolism, regulating the synthesis, catabolism and oxidation of various substances in the body (23), so we used proteomic and metabolomic to examine the overall changes in proteins and metabolites occurring in the liver of simple obese rats. Therefore, this study is proposed to intervene in high-fat diet-fed rats with MSS to investigate the effect on it and explore the specific mechanisms based on proteomics and metabolomics. The results of the study may provide a theoretical basis for the prevention of simple obesity caused by high-fat diet and the search for new healthy dietary patterns.

## Materials and methods

### Animals and experimental studies

The animal study was reviewed and approved by the Laboratory Animal Affairs and approved by the Animal Ethics Committee of Guizhou Medical University. Male Sprague Dawley (SD) rats (weight: 160 ± 20 g) were obtained from the Animal Center of Guizhou Medical University and housed in the animal house of Guizhou Medical University at 20–25°C, 50–70% humidity and 12 h light/dark cycle, and the MSS used for the experiments was purchased from Ming Yang Food Co. Before the start of the intervention, all rats were fed in the animal house for 1 week for acclimatization (normal diet + distilled water). The normal diet and the high-fat were provided by the Animal Center of Guizhou Medical University, and the high-fat diet consisted of 78.8% normal diet, 1% cholesterol, 10% lard, 10% egg yolk powder, and 0.2% bile salt. According to the dietary intake survey, the common dose of MSS is about 10 g/500 mL for adults, and the equivalent dose for rats and humans was converted to determine the MSS intervention dose of 8 g/kg BW per day for this study (Conversion multiplier for 200 g rat to human is 0.16 times) (4). After the start of the intervention, 50 SD rats were stratified by body weight and divided into five groups by random number method as follows (*n* = 10 per group): ND, NDS group were normal diet; HFD, HFDS, HFDIS group were high-fat diet. Intervention: ND group: gavage 8 g/kg BW distilled water for 12 weeks. NDS group: gavage 8 g/kg BW MSS for 12 weeks. HFD group: gavage 8 g/kg BW distilled water for 12 weeks. HFDS group: gavage of 8 g/kg BW MSS for 12 weeks. HFDIS group: gavage of 8 g/kg BW MSS for the first 6 weeks and gavage of 8 g/kg BW distilled water for the last 6 weeks. Five groups of rats were intervened daily at 3 p.m. sharp. The body weight of the rats was measured weekly and at the end of the 12th week, blood samples were collected by intraperitoneal injection of sodium pentobarbital (0.5 mL/100 g BW) after anesthesia, and intra-abdominal fatty tissue and liver tissue were removed and weighed after dissection of the abdomen. Three liver samples from each of the five groups were randomly selected for proteomic assays using the random number method (*n* = 3), and then three more liver samples were randomly selected from the remaining seven samples in each group for metabolomic assays (*n* = 6).

### Tissue measurement index

Lee’s index is a valid index for evaluating the degree of obesity in adult rats (24). The liver index, Lee’s index and adiposity index were calculated using the following equations:


$$\mathrm{Liver}\mathrm{index}\left(\%\right)=\frac{\mathrm{liver}\,\mathrm{weight}\,\left(\mathrm{cm}\right)}{\mathrm{body}\,\mathrm{weight}\,\left(\mathrm{cm}\right)}\times 100$$

$${\mathrm{Lee}}^{\prime }\,s\,\mathrm{index}=\frac{\sqrt[{3}]{\mathrm{body}\,\mathrm{weight}\,\left(g\right)}\times 1000}{\mathrm{nose}\,\text{-to-}\,\mathrm{anus}\,\mathrm{length}\,\left(\mathrm{cm}\right)}$$

$$\mathrm{Adiposity}\mathrm{index}\left(\%\right)\left(\text{25}\right)=\frac{\text{intra}-\mathrm{abdominal}\,\mathrm{fat}\,\left(g\right)}{\mathrm{body}\,\mathrm{weight}\,\left(g\right)}\times 100$$

### Biochemical analysis

Blood was collected into blood collection tubes and left to stand at room temperature for 20 min before centrifugation (3,000 r/min and 4°C) for 15 min using a low-speed benchtop centrifuge (Shanghai Anting Scientific Instruments Factory, TDL-5000bR, Shanghai, China) to obtain serum, then stored at −20°C. The levels of blood lipids, glucose and liver lipid deposition parameters fasting blood glucose (FBG), total glyceride (TG), total cholesterol (TC), low density lipoprotein cholesterol (LDL-C), high density lipoprotein cholesterol (HDL-C) were measured using an automated biochemical analyzer (Beckman Coulter, Lx-20, Brea, USA).

### Histological analysis

Liver tissues were fixed with 10 % paraformaldehyde (Shanghai Maclean Biological Co., Ltd., China) and the tissues were stained with hematoxylin and eosin (H&E) and oil red O (Shanghai Maclean Biological Co., Ltd., China). The results were observed and recorded using a light microscope (20.0x) (Olympus, Tokyo, Japan).

### Quantitative proteomics and analysis by tandem mass tagging

#### Sample processing

The liver samples were taken out from −80°C, weighed the appropriate amount of tissue samples into a pre-cooled mortar with liquid nitrogen, and fully ground to powder with liquid nitrogen adding 4 times the volume of powder lysis buffer and ultrasonically lysed. Then the samples were centrifuged (3,000 r/min and 4°C) for 10 min, and the protein concentration was determined using the bicinchoninic acid (BCA) kit (Beyotime Biotechnology, China). Equal amounts of each sample protein were taken for enzymatic digestion, and the appropriate amount of standard protein was added, and the volume was adjusted to be consistent with the lysis solution. The final concentration of 20% Trichloroacetic acid (Sigma-Aldrich^®^, Shanghai, China) was slowly added, vortexed and mixed, and precipitated at 4°C for 2 h. The precipitate was centrifuged (1,000 r/min and 4°C) for 5 min and washed 2–3 times with pre-chilled Acetone (Zhejiang Hannuo Chemical Technology Co., Ltd., China). After drying the precipitate, add Tetraethylammonium bromide (Sigma-Aldrich^®^, Shanghai, China) at a final concentration of 200 mM, ultrasonically break up the precipitate, add trypsin (Promega Corporation, USA) at a ratio of 1:50 (protease: protein, m/m), and digest overnight. DL-Dithiothreitol (Sigma-Aldrich^®^, Shanghai, China) was added to a final concentration of 5 mM and reduced for 30 min at 56°C. Afterward, iodoacetamide (Sigma-Aldrich^®^, Shanghai, China) was added to a final concentration of 11 mM and incubated for 15 min at room temperature and protected from light.

#### TMT labeling and liquid chromatography-mass spectrometry analysis

Trypsinized peptides were desalted with Strata X C18 (Phenomenex Co., Ltd., USA) and vacuum freeze-dried. The peptides were solubilized and labeled according to the TMT kit (Thermo Fisher Scientific Co., Ltd., China) operating instructions. The simple procedure is as follows: the labeling reagent is thawed and dissolved with Acetonitrile (Thermo Fisher Scientific Co., Ltd., China), mixed with the peptide and incubated at room temperature for 2 h. The labeled peptide is mixed, desalted and vacuum freeze-dried. The peptides were graded by high pH reversed-phase High Performance Liquid Chromatography (HPLC) on an Agilent 300Extend C18 column (5 μm particle size, 4.6 mm inner diameter, 250 mm length). The operation was as follows: the gradient of peptide classification was 8–32% acetonitrile, pH 9, and 60 fractions were separated in 60 min time, and then the peptides were combined into 9 fractions, and the combined fractions were freeze-dried under vacuum for subsequent operation. The peptides were dissolved in liquid chromatography mobile phase A and then separated using an EASY-nLC 1200 ultra-high performance liquid phase system. Mobile phase A was an aqueous solution containing 0.1% formic acid (Fluka Corporation, Shanghai, China) and 2% Acetonitrile; mobile phase B was an aqueous solution containing 0.1% formic acid and 90% Acetonitrile. The liquid phase gradient settings were: 0–26 min, 7–26% B; 26–34 min, 26–38% B; 34–37 min, 38–80% B; 37–40 min, 80% B, with the flow rate maintained at 500 nL/min. The peptides were separated by the Ultra High Performance Liquid Chromatography (UHPLC) system and then injected into the NSI ion source for ionization and then into the Q Exactive™ HF-X mass spectrometer. The ion source voltage was set at 2.1 kV and the peptide parent ions and their secondary fragments were detected and analyzed using a high-resolution Orbitrap (26).

#### Analysis

Based on the Raw files obtained from the mass spectrometry assay, a sample-specific protein database was constructed according to the source of the samples, and then the analysis software was used to search the database; quantitative analysis of the proteins was performed, including quantitative distribution and reproducibility analysis; difference screening was performed according to the quantitative results, and statistical graphs related to different analysis were drawn. The Kyoto Encyclopedia of Genes and Genomes (KEGG) database was used for pathway enrichment analysis of differentially expressed proteins.^1^ Pathway enrichment significance analysis was performed using Fisher’s exact test for differentially expressed proteins (with the identified proteins as background), and *P*-value < 0.05 was considered significant. The ratio of the mean relative quantitative values of each protein in multiple replicate samples was used as the fold of difference (Fold Change, FC), and the relative quantitative values of each protein in the samples were Log2 log-transformed to make the data conform to the normal distribution, and significance analysis was performed using *t*-test. Principal component analysis (PCA) plots were used to show differences between and within groups.

### Metabolomics and analysis

#### Sample processing and assay

The liver samples are removed from the −80°C refrigerator and thawed on ice until they can be cut (all subsequent operations are required to be performed on ice); the sample is chopped and mixed, and 20 mg (±1 mg) is weighed at multiple points into the corresponding numbered centrifuge tube; a steel ball is added with forceps, homogenized for 20 s with a ball mill (30 HZ), and the sample is centrifuged (3,000 r/min and 4°C) for 30 s. After centrifugation, add 400 μL of 70% methanol (Merck) aqueous internal standard extraction solution, shake (1,500 r/min and 4°C) for 5 min, and let stand on ice for 15 min. Centrifuged (12,000 r/min and 4°C) for 10 min, removed 300 μL of supernatant into another corresponding numbered tube, and let stand at −20°C for 30 min. The supernatant was transferred to another tube with the same sample number and left at −20°C for 30 min in the refrigerator. The supernatant was centrifuged (12,000 r/min and 4°C) for 3 min and 200 μL was transferred to the liner tube of the corresponding injection vial for the analysis. The liquid chromatographic conditions were as follows: column: Waters ACQUITY UPLC HSS T3 C18 1.8 μm, 2.1 mm*100 mm; mobile phase A: ultrapure water, 0.1% formic acid (Thermo Fisher Scientific Inc., Shanghai, China); mobile phase B: acetonitrile, 0.1% formic acid; column temperature: 40°C; flow rate: 0.40 mL/min; injection volume: 2 μL. The injection volume: 2 μL.

#### Analysis

The raw data from the mass spectrometry downcomers were converted to mzML format by ProteoWizard, and the XCMS program was used for peak extraction, alignment, and retention time correction. Peak areas were corrected by the “SVR” method, and peaks with > 50% deletion were filtered for each group of samples. After correction and filtering, the peaks were identified by searching the database, integrating the public library and metDNA method to obtain metabolite identification information. The metabolite content data were standardized by unit variance scaling (UV), and the accumulation pattern of metabolites among different samples was analyzed by cluster analysis (Hierarchical cluster analysis, HCA) through R software v3.5.0. The ratio of the mean relative quantitative values of each metabolite in multiple replicate samples was used as the fold change (FC), and then the relative quantitative values of each metabolite in the comparison group samples were subjected to *t*-test to calculate the corresponding *P*-value, which was used as the significance index. A combination of FC, *P*-value, and projection value of Variable Importance Projection (VIP) of OPLS-DA model was adopted to screen the differential metabolites. Screening criteria: (1) Metabolites with FC ≥ 2 and FC ≤ 0.5 were selected. Metabolites with FC ≥ 2 and FC ≤ 0.5 were considered to be significantly different between the control and experimental groups. (2) Metabolites with *P*-value < 0.05 were selected. The metabolites with statistically significant differences between groups were considered significant. (3) Metabolites with VIP ≥ 1 were selected. VIP value indicates the intensity of the group differences of the corresponding metabolites in the model in the classification of each group of samples, and metabolites with VIP ≥ 1 were generally considered significant. Pathway enrichment significance analysis was performed for differential metabolites using Fisher’s exact test, and *P*-value < 0.05 was considered significant. PCA plots were used to show differences between and within groups.

### Combined multi-omics analysis

The list of differential proteins (Supplementary Data Sheet 1) and differential metabolites (Supplementary Data Sheet 2) was uploaded to metaboanalyst 5.0^2^ for KEGG pathway analysis and Cytoscape was used to map the protein-metabolite interaction network.

### Statistical analysis

SPSS22.0 software was used to analyze the differences between groups in terms of body weight, tissue measurement index, the levels of blood lipids and FBG. When the data showed normal distribution, one-way ANOVA was used for homogeneity of variance followed by the least significant difference test to compare means among groups. Differences with *P* < 0.05 were considered significant.

## Results

### Body weight and body fat deposition

**FIGURE 1:**
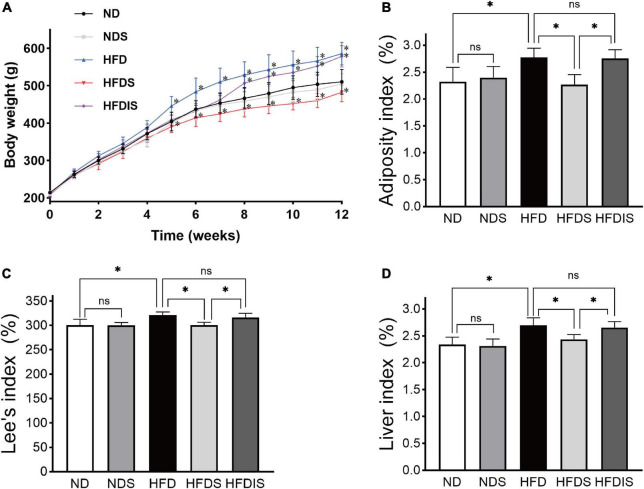
Effect of Miao sour soup (MSS) on the Body Weight and Fat Deposition of high-fat diet (HFD)-fed rats. **(A)** Body weights of rats during the 12 weeks. **(B)** Adiposity index of rats. **(C)** Lee’s index of rats. **(D)** Liver index of rats. ND: normal diet + distilled water (12 weeks); NDS: normal diet + MSS (12 weeks); HFD: high-fat diet + distilled water (12 weeks); HFDS: high-fat diet + MSS (12 weeks); HFDIS: high-fat diet + MSS (the first 6 weeks) and then distilled water (the last 6 weeks). *p < 0.05 indicates significant difference, ns indicates no significant difference (ND vs. NDS, HFD; HFD vs. HFDS, HFDIS; HFDS vs. HFDIS).

We evaluated the effect of MSS on body weight and body fat deposition in rats fed a high-fat diet, as shown in Figures 1A–D. Before the start of the experiment, there was no difference in body weight between the five groups (*P* > 0.05), at the end of the 12th week. The body weight was higher in the HFD group compared to the ND group (*P* < 0.05), there was no difference in body weight in the ND group compared to the NDS group (*P* > 0.05), the body weight in the HFDS group was lower than that in the HFD group and HFDIS group (*P* < 0.05), and there was no difference in body weight between the HFDIS and HFD groups (*P* > 0.05). In addition, the adiposity index and Lee’s index and liver index were higher in the HFD group than in the ND and HFDS groups (*P* < 0.05) and did not differ from the HFDIS group (*P* > 0.05), and were lower in the HFDS group than in the HFDIS group (*P* < 0.05). There was no difference between the ND and NDS groups (*P* > 0.05). The results showed that the intervention of MSS reduced body weight gain and body fat deposition in high-fat diet-fed rats.

### Blood lipids, glucose and liver lipid deposition

**FIGURE 2:**
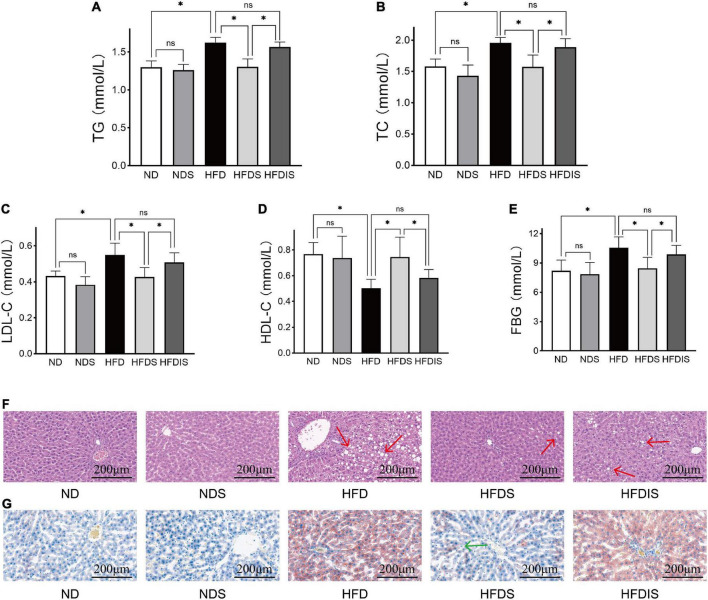
Effect of MSS on the serum lipid levels, fasting blood glucose (FBG) and the fat formation in the liver of HFD-Fed rats. **(A)** Triglyceride (TG). **(B)** Total cholesterol (TC). **(C)** Low- density lipoprotein cholesterol (LDL- C). **(D)** High-density lipoprotein cholesterol (HDL- C). **(E)** Fasting blood glucose. **(F)** Representative images of HE staining of rat livers, red arrows point to fat vacuoles. **(G)** Representative images of Oil Red O staining of rat livers, green arrow point to fat deposition. *p < 0.05 indicates significant difference, and ns indicates no significant difference.

We examined the changes of serum lipid levels and FBG in obese rats to assess the effects of MSS on glycolipid metabolism. Figures 2A–E shows the results of TG, TC, LDL-C, HDL-C, and fasting glucose (FBG) in each group, as shown in the figure, serum TG, TC, LDL-C, and FBG in the HFD group were higher than those in the ND group (*P* < 0.05), and HDL-C concentration decreased (*P* < 0.05). Serum TG, TC, LDL-C, and FBG were lower in the HFDS group than in the HFD group (*P* < 0.05), and HDL-C increased (*P* < 0.05). The results of TG, TC, LDL-C, HDL-C, and FBG were not different between the ND and NDS groups and between the HFDIS and HFD groups (*P* > 0.05).

The results of liver H&E (Figure 2F) and oil red O (Figure 2G) staining showed that the hepatocytes in the ND and NDS groups were normal in shape and neatly arranged, and no significant abnormalities were observed. HFDIS group switched to distilled water gavage after 6 weeks of MSS gavage intervention, and HFDS group completed the full 12 weeks intervention. HFDIS group was similar to the HFD group, with obvious cellular fatty degeneration and round vacuoles of different sizes in the cytoplasm, suggesting that the whole intervention of MSS could effectively improve liver fat deposition.

### Differences in liver protein expression

**FIGURE 3:**
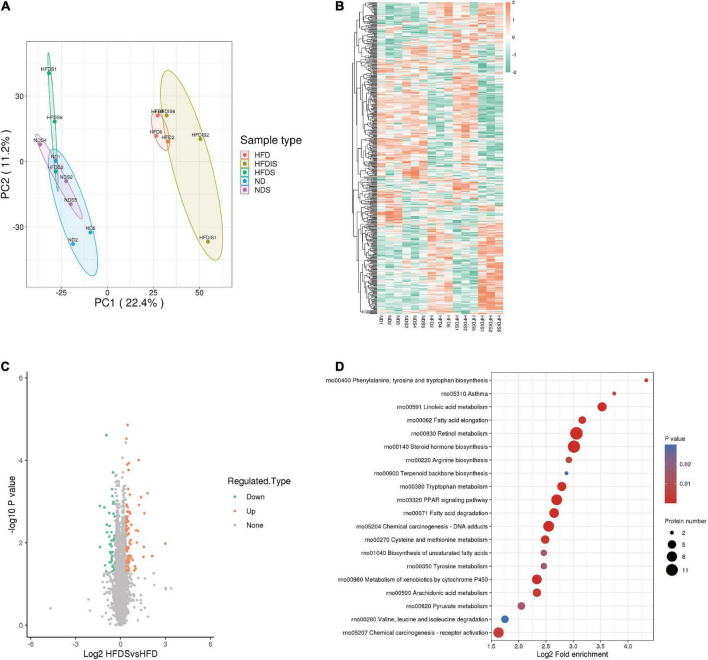
Differentially expressed proteins (DEPs) were identified and analyzed using proteomics in the liver of HFD-Fed rats. **(A)** Results of quantitative protein principal component analysis (PCA) of all liver samples. **(B)** Cluster chart of DEPs. Red and green represent significant upward and downward adjustments, respectively. **(C)** Volcano map for the HFDS vs. HFD group. The red and green dots represent significantly up- and down-regulated differential proteins, respectively. The black dots represent proteins that showed no difference. **(D)** Bubble plot of the top 20 KEGG enrichment pathways of DEPs. Fold enrichment is the Log2-transformed value of the fold change in the proportion of differentially expressed proteins in that functional type compared to the proportion of identified proteins.

In order to reveal the specific mechanism of action of MSS on simple obese rats caused by high-fat diet feeding, we performed TMT quantitative proteomic analysis on the livers of five groups of rats. The principle component analysis (PCA) showed the differences between and within groups (Figure 3A). A total of 7,218 proteins were identified in this result. The results of the clustering heat map showed the differences in the expression of different proteins among the groups (Figure 3B), with red indicating up-regulation and green indicating down-regulation. Among them, 76 proteins were up-regulated and 50 proteins were down-regulated in the HFDS group compared with the HFD group (Figure 3C). The KEGG pathway analysis showed that these differential proteins were mainly involved in biological processes such as lipid metabolism and amino acid metabolic reactions (Figure 3D).

A total of 33 protein expressions were upregulated in the HFDS group compared with the HFD group and downregulated in the HFD group compared with the ND group; a total of 19 protein expressions were downregulated in the HFDS group compared with the HFD group and upregulated in the HFD group compared with the ND group (Table 1).

**TABLE 1:** Fifty-two differentially expressed proteins (DEPs) common to three groups (ND, HFD, HFDS).

Protein accession	Gene name	HFDS/HFD			HFD/ND		
			
		Type	Ratio	*P*-value	Type	Ratio	*P*-value
Q62967	Mvd	Down	0.614	0.0168833	Up	2.01	0.0043481
P07379	Pck1	Down	0.707	0.0001971	Up	1.49	0.0009863
A4F267	Tomm40l	Down	0.591	0.0019055	Up	1.32	0.0075392
G3V9G4	Acly	Down	0.633	0.0180745	Up	1.534	0.0444326
D4A605	Nnmt	Down	0.606	0.0110026	Up	1.564	0.0141326
D4A904	Nags	Down	0.711	0.0011298	Up	1.361	0.003964
A0A0G2JSK9	Bhmt	Down	0.474	0.0013865	Up	1.855	0.0053504
D4A147	Ugt2a3	Down	0.591	0.007114	Up	1.68	0.0134986
P13221	Got1	Down	0.672	0.0005016	Up	1.652	0.0044859
P12785	Fasn	Down	0.531	0.0036612	Up	2.195	0.00184
P09034	Ass1	Down	0.686	0.0007396	Up	1.41	0.003824
Q499N5	Acsf2	Down	0.384	0.0127379	Up	2.508	0.0085134
P11497	Acaca	Down	0.741	0.007918	Up	1.5	0.0033102
Q4KLP0	Dhtkd1	Down	0.519	2.454E-05	Up	1.786	0.0006555
Q9Z2Z8	Dhcr7	Down	0.689	0.0382657	Up	1.833	0.0020237
P55053	Fabp5	Down	0.512	0.0493997	Up	2.176	0.0169945
P85968	Pgd	Down	0.664	0.0121654	Up	1.627	0.0091824
P13255	Gnmt	Down	0.732	0.0401861	Up	1.411	0.0032176
Q9JI61	Lrat	Down	0.53	0.0055678	Up	1.695	0.0204272
A0A0G2JTY8	Tnip3	Up	1.587	0.0211375	Down	0.629	0.0022106
A0A0G2JW22	–	Up	1.452	0.0044365	Down	0.693	0.0029882
P55159	Pon1	Up	1.537	0.0006797	Down	0.64	0.0002279
P04916	Rbp4	Up	1.338	0.0048538	Down	0.671	0.0278837
P17764	Acat1	Up	1.336	0.0026633	Down	0.75	0.0038604
P07871	Acaa1b	Up	2.043	0.005032	Down	0.601	0.0486322
B2GV28	Cyp2b1	Up	2.25	0.0051696	Down	0.618	0.0102066
Q4KLZ0	Vnn1	Up	2.315	9.9E-05	Down	0.45	0.0006793
A0A0G2K7Z3	Acot1	Up	1.433	0.0208687	Down	0.626	0.0024711
Q6AXY8	Dhrs1	Up	1.647	0.0036863	Down	0.556	0.0047469
Q32Q55	Ces2h	Up	1.326	0.0051577	Down	0.754	0.0005085
G3V8J2	Cyp8b1	Up	1.39	0.0064143	Down	0.756	0.0035514
G3V734	Decr1	Up	1.446	0.0001717	Down	0.672	8.014E-05
D3Z899	Miga2	Up	1.359	0.0152525	Down	0.761	0.033144
D3ZIQ1	Acot4	Up	1.358	0.0017493	Down	0.704	0.0034159
A0A0G2K5K4	Cyp3a62	Up	1.465	0.0001155	Down	0.678	0.0025087
O70597	Pex11a	Up	1.404	0.0236595	Down	0.677	0.016332
P19225	Cyp2c70	Up	1.379	0.0029429	Down	0.76	0.0325922
P05183	Cyp3a2	Up	2.112	0.0098944	Down	0.58	0.0218882
Q6AYS8	Hsd17b11	Up	1.421	0.0004965	Down	0.47	0.0002959
P15149	Cyp2a2	Up	1.326	0.0458872	Down	0.713	0.0150033
P08516	Cyp4a10	Up	1.577	0.0109137	Down	0.524	0.0031558
P20816	Cyp4a2	Up	3.011	0.0019259	Down	0.333	0.0376205
Q62651	Ech1	Up	1.439	0.0017779	Down	0.693	0.0001926
Q64428	Hadha	Up	1.356	0.0001274	Down	0.688	0.0002016
P00185	Cyp1a1	Up	1.452	0.0134213	Down	0.729	0.0246289
P55051	Fabp7	Up	2.579	0.0007857	Down	0.527	0.0015247
Q60587	Hadhb	Up	1.38	1.392E-05	Down	0.685	3.058E-05
P07687	Ephx1	Up	1.593	0.0086234	Down	0.73	0.0053139
P51647	Aldh1a1	Up	3.498	0.0006259	Down	0.402	0.0173614
Q9Z2M4	Decr2	Up	1.493	0.0204946	Down	0.767	0.0161494
B2GV54	Nceh1	Up	1.348	0.0163398	Down	0.748	0.0032946
D3ZE56	Pik3c2g	Up	1.876	0.0473421	Down	0.676	0.0328512

### Differences in liver metabolites

**FIGURE 4:**
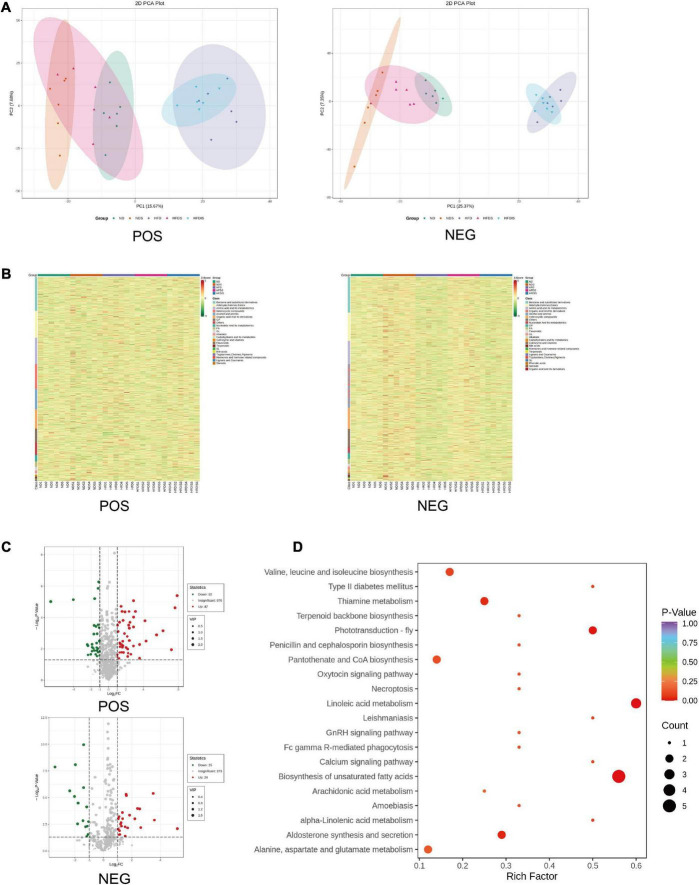
Differential metabolites were identified and analyzed using metabonomics in the liver of HFD-Fed rats. POS indicates metabolites with positive ions in the mass spectrum, NEG indicates metabolites with negative ions in the mass spectrum. **(A)** Results of metabolites principal component analysis (PCA) of all liver samples. **(B)** Cluster chart of differential metabolites. Red and green represent significant upward and downward adjustments, respectively. **(C)** Volcano map for the HFDS vs. HFD group. The red and green dots represent significantly up- and down-regulated differential metabolites, respectively. **(D)** Bubble plot of the top 20 KEGG enrichment pathways of differential metabolites. Rich Factor is defined as the number of differential metabolites annotated to the pathways divided by all identified metabolites annotated to the pathway.

In order to reveal the specific mechanism of action of MSS on simple obese rats caused by high-fat diet feeding, we further performed metabolomic analysis on the liver of five groups of rats. The PCA showed the differences between and within groups (Figure 4A). We identified a total of 4,961 metabolites in this experiment, which resulted in 2,369 for Positive ions (POS) and 2,592 for Negative ions (NEG), and the results of the clustering heat map showed the differences of different metabolites among the groups (Figure 4B), with red indicating up-regulation and green indicating down-regulation. In the HFDS group, 47 metabolites were up-regulated and 71 metabolites were down-regulated compared with the HFD group (Figure 4C). KEGG pathway analysis of these differential metabolites mainly involved biological processes such as fatty acid metabolism and amino acid metabolic reactions (Figure 4D).

A total of nine metabolite contents were upregulated in the HFDS group compared with the HFD group and downregulated in the HFD group compared with the ND group; a total of 10 metabolite contents were downregulated in the HFDS group compared with the HFD group and upregulated in the HFD group compared with the ND group (Table 2).

**TABLE 2:** Nineteen differential metabolites common to three groups (ND, HFD, HFDS).

Index	Compounds	Formula	HFDS/HFD				HFD/ND			
				
			Type	VIP	Fold change	*P*-value	Type	VIP	Fold change	*P*-value
MW0054374	Linoleic acid	C18H32O2	Down	1.662	0.383	0.0000000	Up	1.753	2.376	0.0000000
MW0052792	FA 18:0	C18H36O2	Down	1.623	0.095	0.0000000	Up	1.608	7.425	0.0000000
MW0015854	Arachidonic acid	C20H32O2	Down	1.657	0.245	0.0000078	Up	1.727	3.436	0.0000115
MW0052998	Gamma-Linolenic acid	C18H30O2	Down	1.342	0.420	0.0053951	Up	1.200	2.113	0.0113119
MEDN0400	FFA (18:3)	C18H30O2	Down	1.641	0.194	0.0000023	Up	1.636	3.597	0.0000006
MEDP0437	Ergothioneine	C9H15N3O2S	Down	1.927	0.437	0.0000014	Up	2.126	2.253	0.0000050
MW0140553	(Indol-3-yl)glycolaldehyde;3-Indoleglycolaldehyde	C10H9NO2	Down	1.382	0.269	0.0139956	Up	1.078	2.242	0.0456326
MW0017083	Cholesterol 3-sulfate	C27H46O4S	Down	1.526	0.286	0.0028630	Up	1.605	4.550	0.0018005
MW0003976	3-Ethylbenzene-1,2-diol	C8H10O2	Down	1.263	0.386	0.0127729	Up	1.294	2.426	0.0140246
MW0116699	1,4-Dihydro-1-methyl-4-oxo-3-pyridinecarboxamide	C7H8N2O2	Down	1.349	0.387	0.0083116	Up	1.521	3.506	0.0028496
MEDN0204	Pyruvic Acid	C3H4O3	Up	1.280	2.420	0.0046872	Down	1.325	0.367	0.0081477
MW0126762	Thiamine monophosphate	C12H18N4O4PS	Up	1.468	3.577	0.0004368	Down	1.529	0.358	0.0001054
MW0154120	N,N-Dihydroxy-L-valine	C5H11NO4	Up	1.609	4.622	0.0009570	Down	1.669	0.267	0.0006418
MW0105712	Argininosuccinic acid	C10H18N4O6	Up	1.812	4.014	0.0082129	Down	1.622	0.337	0.0205920
MW0010027	Valine	C5H11NO2	Up	1.725	2.988	0.0079625	Down	1.734	0.365	0.0126669
MW0126862	Trichloroepoxyethane	C2HCl3O	Up	1.485	3.261	0.0068081	Down	1.543	0.305	0.0036668
MW0124660	Leucodopachrome	C9H9NO4	Up	1.816	3.092	0.0032985	Down	1.905	0.339	0.0016535
MEDN1130	NADH	C21H29N7O14P2	Up	1.445	6.154	0.0063602	Down	1.553	0.242	0.0063693
MEDP0039	Betaine	C5H11NO2	Up	1.830	4.592	0.0103242	Down	1.698	0.307	0.0158143

### Combined multi-omics analysis

**FIGURE 5:**
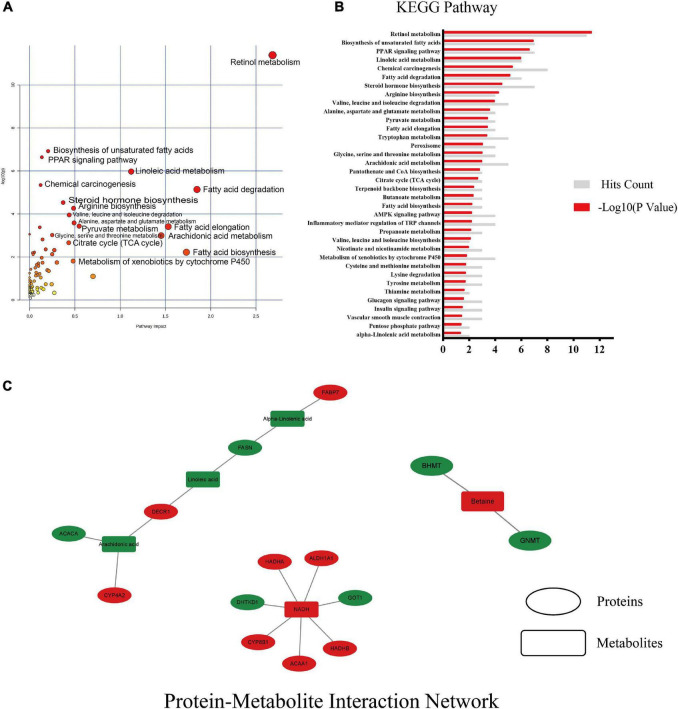
Results of combined multi-omics analysis. **(A)** KEGG pathway with pathway impact and significance. Pathway impact is a combination of the centrality and pathway enrichment results, it is a tool to measure centrality or “hubness” in an objective manner. **(B)** KEGG enrichment pathway with significant differences and the number of hits on that pathway. **(C)** protein-metabolite interaction network of differential proteins and differential metabolites, red indicates up-regulation after MSS intervention, green indicates down-regulation. Ellipses indicate proteins and rectangles indicate metabolites, red indicates up-regulation after MSS intervention, green indicates down-regulation after MSS intervention.

The data of 52 differential proteins and 19 differential metabolites were uploaded to the metaboanalyst 5.0 to analyze the effect of the whole intervention of MSS in the liver of rats fed with high-fat diet and its mechanism (Figures 5A,B). The protein-metabolite interaction network was mapped using Cytoscape (Figure 5C), and the results showed that the protein and downstream metabolites were mainly enriched to the PPAR signaling pathway, Retinol metabolism, Fatty acid β-oxidation, Fatty acid degradation, and Fatty acid biosynthesis, Glycine, serine and threonine metabolism, Pyruvate metabolism, Citrate cycle (TCA cycle), and other pathways.

The proteins that are up-regulated in the aforementioned pathways are as follows: retinal dehydrogenase (ALDH1A), fatty acid-binding protein 5 (FABP5), acetyl-CoA acyltransferase 1 (ACAA1), 2,4-dienoyl-CoA reductase (DECR2), 3,5-tetradecadienoyl-CoA isomerase (ECH1), and peroxin-11A (PEX11A), acetyl-CoA acyltransferase 2 (ACAA2), enoyl-CoA hydratase (HADHA), enoyl-CoA hydratase (ECHS1). The proteins that are down-regulated in the aforementioned pathways are as follows: acetyl-CoA carboxylase (ACACA), fatty acid synthase (FASN), betaine-homocysteine S-methyltransferase (BHMT), glycineN-methyltransferase (GNMT), phosphoenolpyruvate carboxykinase (PCK1), ATP citrate (pro-S)-lyase (ACLY). The metabolites that are up-regulated in the aforementioned pathways are as follows: reduced nicotinamide adenine dinucleotide (NADH), Betaine and Pyruvate. The metabolites that are down-regulated in the aforementioned pathways are as follows: fatty acids (linoleic, arachidonic, γ-linolenic, α-linolenic, and octadecanoic acid).

It is suggested that the intervention of MSS may promote metabolism through the above biological responses and thus improve metabolic disorders and prevent the development of simple obesity in rats fed with high fat diet.

## Discussion

**FIGURE 6:**
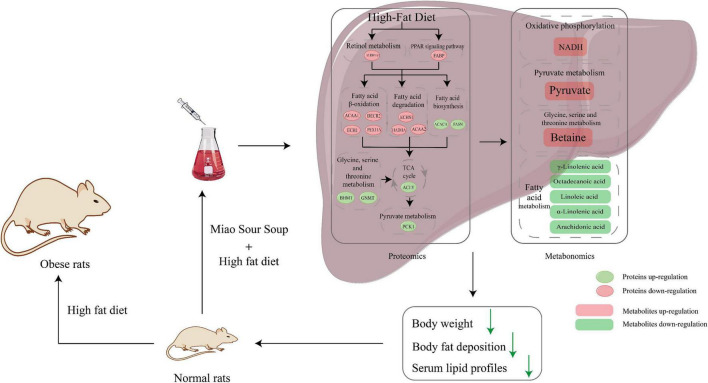
Schematic diagram of Miao Sour Soup to improve energy metabolism in the liver of HFD-Fed rats. Ellipses indicate proteins and rectangles indicate metabolites, red indicates up-regulation after MSS intervention, green indicates down-regulation after MSS intervention.

Figure 6 is an overview graph of the results of the study, in which rats with simple obesity induced by high-fat diet feeding for 12 weeks showed significantly lower body weight, FBG, blood lipids, and body fat deposition levels, and significantly improved hepatic steatosis and lipid deposition after MSS intervention compared with rats after simultaneous feeding of MSS and high-fat diet for 12 weeks (Figures 1, 2). We also set up an NDS group to demonstrate that MSS does not have adverse health effects on rats on a normal diet (ND group). Interestingly, we found that the rats that stopped the intervention after the MSS half-time intervention (HFDIS group) had similar results to those of the HFD group for each index. It is suggested that the sustained intervention of MSS significantly improved the disorder of body lipid metabolism caused by high-fat diet, but it is possible that the dietary intervention needs to be continued. The results of proteomics and metabolomics showed that the MSS intervention promoted lipid oxidation in the liver, reduced lipid synthesis, and caused a large amount of energy to be consumed by fatty acid metabolism in the liver, thus restoring the normal level of fatty acid content in the liver due to excessive accumulation of high-fat diet. At the same time, the intervention of MSS also regulated the process of pyruvate metabolism and other biological processes in rats fed with the high-fat diet, further improving the metabolic disorder process in high-fat diet rats.

Lipids play an important role in the life activities of the body, directly participating in the regulation of cellular signaling pathways and gene transcription, as well as being an important energy substrate in the body (27). Dietary fatty acids are emulsified in the intestine by bile acids into fine microaggregates that are absorbed by intestinal mucosal cells and packaged as celiac particles that are transported to the liver (28). In hepatocytes, fatty acids are mainly transported to the mitochondria for β-oxidation to produce acetyl CoA, which enters the citric acid cycle and then is completely oxidized to release a large amount of energy for the body. The excess fatty acids are processed into triglycerides or cholesteryl esters, which are stored in lipid droplets (LD) or formed as VLDL secreted into the blood and transported to extrahepatic tissues for action (29). The PPAR signaling pathway plays a key role in the metabolism of lipids, regulating downstream genes involved in lipid oxidation and metabolism (30), while retinol metabolic processes are related to sterol and triglyceride metabolism (31). FABP5 in the PPAR signaling pathway is a small molecule intracellular lipid chaperone that binds to hydrophobic ligands such as long-chain fatty acids and transports them to organelles such as mitochondria for further metabolism (32), mitochondria are key organelles for energy metabolism, ALDH1A1 is responsible for the oxidation of retinaldehyde to retinoic acid during retinol metabolism, and studies have shown that retinoic acid plays a key role in mitochondrial β-oxidation (31), and their upregulation promotes lipid metabolic processes. The key proteins in fatty acid β-oxidation, ACAA1, PDCR2, ECH1, Pex11a, ACAA2, HADHA, and ECHS1, were upregulated. ACAA1, PDCR2, ECH1, and PEX11A are important proteins in the peroxisome pathway and play a central role in lipid metabolism, the PEX11A is a key enzyme in the peroxisome and its deficiency impairs peroxisome abundance and fatty acid β-oxidation and leads to hepatic triglyceride accumulation (33). ACAA1 is widely present in humans and animals and catalyzes the synthesis of esterified cholesterol from free cholesterol and long-chain fatty acids (34). PDCR2 is a key enzyme for β-oxidation of polyunsaturated fatty acids (PUFAs), and studies have shown that knockdown of PDCR2 leads to reduced oxidation of unsaturated fatty acids, allowing lipid accumulation (35). Ech1 is also involved in fatty acid β-oxidation in mitochondria (36). ECHS1, HADHA, and ACAA2 are key enzymes in fatty acid β-oxidation, catalyzing the eventual conversion of Hexadecanoyl-CoA to Acetyl-CoA, and studies have shown that ECHS1 enzyme deficiency leads to lead to impaired ATP production as well as fatty acid accumulation (37), HADHA overexpression reduces cytoplasmic LD formation (38), while ACAA2 is involved in mitochondrial fatty acid elongation and degradation by catalyzing the last step of the respective β-oxidation pathway (39), and its overexpression inhibits triglyceride production (40). On the other hand, intervention with MSS reduced the expression of FASN, ACACA, which is involved in fatty acid synthesis, and ACLY in the tricarboxylic acid cycle in the liver of simple obese rats. FASN is a key enzyme for the ab initio synthesis of fatty acids and uses acetyl coenzyme A as a primer, malonyl coenzyme A as a two-carbon donor and NADPH as a reducing equivalent to synthesize long-chain fatty acids (41). ACACA can catalyze the carboxylation of acetyl coenzyme A to form malonyl coenzyme A, which is the rate-limiting and key regulatory step in the ab initio synthesis of fatty acids (42). It is currently believed that inhibition of acetyl coenzyme A carboxylase as a means to develop drugs may be useful in the treatment of patients with nonalcoholic fatty liver disease (NAFLD) (43). In contrast, ACLY in the tricarboxylic acid cycle catalyzes the conversion of citric acid to oxaloacetate and acetyl coenzyme A, which then promotes the synthesis of fatty acids, cholesterol and acetylcholine, it will be abnormally expressed when metabolism is disturbed (44).

Further results of metabolomics showed an increase in the content of NADH in the liver, as a key energy transfer intermediate, which is produced from NAD+ during the oxidation of energy substrates (45). At the same time, the content of fatty acids was reduced, mainly linoleic, arachidonic, γ-linolenic, α-linolenic, and stearic acids. Linoleic acid is a precursor of arachidonic acid, which produces pro-inflammatory eicosanoids and endogenous cannabinoids (46). γ-linolenic acid may have a preventive role in the treatment of various chronic disease states (47), flaxseed oil rich in plant-derived α-linolenic acid improves polycystic ovary syndrome through the steroid hormone-microbiota-inflammation axis (48), and stearic acid (FA 18:0) can lower low-density lipoprotein cholesterol (LDL-C) (49). A high-fat diet leads to a disturbance of lipid metabolism in the body with an abnormal increase in fatty acid content, inducing a significant inflammatory response in adipose tissue and secreting inflammatory markers such as tumor necrosis factor α into the body to further aggravate obesity (50). The results of proteomics and metabolomics showed that the intervention of MSS promoted the lipid metabolism in the liver of rats, consumed the excessive fatty acid oxidation to produce energy brought by the high-fat diet, restored the fatty acid content to the level of normal diet, and improved the disorder of lipid metabolism in the body caused by the high-fat diet.

Moreover, the proteomic results also revealed reduced expression of PCK1 during pyruvate metabolism and BHMT, GNMT during Glycine, serine and threonine metabolism. PCK1 converts oxaloacetate to phosphoenolpyruvate, which further generates glucose via the gluconeogenesis process and is a key enzyme in gluconeogenesis, especially in the liver and kidney, when overexpression of PCK1 leads to increased glucose output and worsening of diabetes, while deletion of PCK1 leads to fatal hypoglycemia (51). The downregulation of pck1 may lead to the accumulation of pyruvate. Pyruvate is a key substance in the process of energy metabolism. In mitochondria, pyruvate and NAD+ are converted to acetyl coenzyme A, NADH and carbon dioxide, and finally to ATP by oxidative phosphorylation, and acetyl coenzyme A and oxaloacetate are transported to the cytoplasm by the action of citrate synthase to produce citric acid for the resynthesis of pyruvate (52). BHMT and GNMT are key proteins in the metabolism of betaine to glycine, and downregulation results in increased levels of betaine. Betaine has been shown to reduce liver fat accumulation in rats, improve insulin resistance, and reduce hepatic steatosis (53). The intervention of MSS may regulate pyruvate metabolism by limiting PCK1, a key enzyme in the process of pyruvate metabolism, and limit the process of gluconeogenesis, while increasing the amount of betaine in the liver, improving the metabolism in the liver of rats fed with the high-fat diet.

## Conclusion

In conclusion, it is suggested that MSS intervention may promote lipid metabolism in the liver of simple obese rats through the above-mentioned biological processes. It may improve metabolic disorders in the liver and restore it to the level to some extent of rats feeding with a normal diet by decreasing lipid deposition, and regulating the process of pyruvate metabolism and other biological processes and regulating the process of pyruvate metabolism and other biological processes. MSS is a traditional food which is the main components of Guizhou diet pattern. It has been consumed for more than 100 years in the Qiandongnan region of Guizhou Province, China. It was found in this study that MSS may have a preventive effect on simple obesity caused by high-fat diet. The results of this study may provide direction and theoretical basis for finding new healthy dietary patterns for obesity prevention, and we will further verify this through *in vitro* experiments and population surveys.

## Data availability statement

The datasets presented in this study can be found in online repositories. The names of the repository/repositories and accession number(s) can be found in the article/Supplementary material.

## Ethics statement

The animal study was reviewed and approved by the Laboratory Animal Affairs and approved by the Animal Ethics Committee of Guizhou Medical University.

## Author contributions

QY: conceptualization (equal), data curation (equal), formal analysis (equal), investigation (equal), methodology (equal), resources (equal), visualization (lead), software (equal), and writing – original draft (lead). QZ: conceptualization (equal), data curation (equal), formal analysis (equal), investigation (equal), methodology (equal), and resources (equal). NW: supervision (equal), validation (equal), and writing – review and editing (equal). YZ, HH, and SH: investigation (equal) and methodology (equal). HW: conceptualization (equal), funding acquisition (equal), methodology (equal), project administration (equal), supervision (equal), validation (equal), and writing – review and editing (lead). All authors contributed to the article and approved the submitted version.

## Footnotes

1
https://www.kegg.jp/

2
https://www.metaboanalyst.ca/
